# Highly Sensitive Loop-Mediated Isothermal Amplification for the Detection of *Leptospira*

**DOI:** 10.1155/2015/147173

**Published:** 2015-01-27

**Authors:** Hua-Wei Chen, Giulia Weissenberger, Erin Atkins, Chien-Chung Chao, Yupin Suputtamongkol, Wei-Mei Ching

**Affiliations:** ^1^Naval Medical Research Center, Silver Spring, MD 20910, USA; ^2^Uniformed Services University of the Health Sciences, Bethesda, MD 20814, USA; ^3^Siriraj Hospital, Mahidol University, Bangkok 10700, Thailand

## Abstract

Leptospirosis is a worldwide zoonosis caused by an infection with the pathogenic species of *Leptospira*. We have developed a loop-mediated isothermal amplification (LAMP) assay to detect the DNA of *Leptospira* spp. Six sets of primers targeting the gene of the subsurface protein, *lipL32*, were evaluated for their detection sensitivity. The best primer set detected less than 25 copies of *lipL32* per reaction of both plasmid DNA template and purified leptospiral genomic DNA. By combining primers targeting *lipL32* with the previously published primer set targeting *lipL41*, the sensitivity of the assay was improved to 12 copies of *L. interrogans*. The specificity of the LAMP assay was evaluated by using the genomic DNA from other clinically encountered bacterial species such as different strains of *Orientia tsutsugamushi*, *Rickettsia typhi*, *Rickettsia conorii*, *Rickettsia rickettsii*, *Coxiella burnetii*, and *Bartonella bacilliformis*. These genomic DNA samples were all negative in our LAMP assay. The sensitivity of the LAMP assay was very similar to that of quantitative real time PCR. Several detection methods for the amplified product of LAMP assay were performed to demonstrate the simplicity of the assay. In summary, our results have suggested that this assay is rapid, robust, and easy to perform and has the potential to be used in endemic locations.

## 1. Introduction

Leptospirosis is considered to be the most widespread zoonosis [1]. This worldwide emerging infectious disease is caused by the pathogenic species belonging to the genus* Leptospira*. More than 1.7 million cases of severe leptospirosis are reported each year, with a case mortality rate of about 10% [2]. It is an underrecognized health problem especially in developing countries. Untreated leptospirosis can eventually lead to hepatorenal failure, pulmonary hemorrhage syndrome, and even death depending on bacterial virulence and the host immune response [3, 4]. The clinical symptoms are similar to a variety of other infectious diseases which are often prominent in the same geographic regions, including scrub typhus, dengue, and malaria [5]. Although the infection can be cured with proper antibiotic therapy at the onset of the disease, the lack of rapid diagnostic tests often presents a barrier to early diagnosis [6].

Microscopic agglutination test (MAT) is considered the “gold standard” of serologic diagnosis. This assay requires ample time as it works by detecting the antibody titer increase in serum samples obtained weeks apart. While this technique provides an efficient retrospective diagnosis, it does not provide early diagnosis [7]. Other diagnostic tests include dark-field microscopy, enzyme-linked immunosorbent assay, and Western blot and are known to have low sensitivity [8]. Polymerase chain reaction (PCR) and quantitative real time PCR (qPCR) can be used for detecting leptospirosis in clinical samples within the first week of illness with low clinical sensitivity [3]. Only about 50% of culture and/or MAT confirmed cases were detected positive by qPCR [9]. Both PCR and qPCR are costly and often not readily available in many laboratories, especially those where leptospirosis is endemic.

Originally described by Notomi et al. [10], loop-mediated isothermal amplification (LAMP) offers an alternative DNA amplification method. LAMP uses* Bst* DNA polymerase for strand displacement DNA synthesis along with primers that create cauliflower-like structures with multiple loops [10]. The most significant advantage of LAMP is that amplification occurs under isothermal conditions. Therefore, only a heating block or an incubator is required. Visualization of amplified DNA products on gel electrophoresis is the most accurate method which can differentiate true positives from false positives due to nonspecific amplification. However the procedures involved in gel electrophoresis are not practical for resource-limited areas. The reaction products can be seen by several alternative methods which are easy to perform, such as turbidity derived from magnesium pyrophosphate formation [11], using a fluorescent dye such as SYBR green to be visualized under UV light [12] or by metal indicators such as hydroxy naphthol blue (HNB) or calcein, which can be seen by the naked eye [11, 13].

We developed a loop-mediated isothermal amplification assay using a combination of primer sets targeting the* lipL32* and* lipL41* genes.* lipL32* encodes a major conserved subsurface protein [14] and is expressed in all pathogenic* Leptospira* [15]. The* lipL41* gene is conserved in 15 serogroups of* L. interrogans* [16]. In this study, we optimized reactions for* Leptospira *spp. detection, examined the specificity and limit of detection of the method, and implemented the method to mimic blood samples. Our results demonstrate the assay's potential use in* Leptospira* endemic areas where resource setting is limited.

## 2. Materials and Methods

### 2.1. Design of Primers

Oligonucleotide primers used for LAMP were based on the* lipL32* genes of* L. interrogans* serovar Copenhageni strain Fiocruz L1-130 (ATCC, VA). Six sets of primers targeting* lipL32* were designed by Primer Explorer V4 (http://primerexplorer.jp/e/). The primer set used for* lipL41* was the same as the one previously described by Lin et al. [17]. All primers were synthesized by Eurofins MWG Operon (Huntsville, AL) and the final choice of primers is described in Table 1.

### 2.2. Plasmid and Genomic DNA Template

The* lipL32* and* lipL41* genes from* L. interrogans* serovar Copenhageni strain Fiocruz L1-130 (ATCC, VA) were cloned into a pET28a vector (Novagen, CA). The closed circular plasmids (pET28a-lipL32 (pL32) and pET28a-lipL41 (pL41)) were purified using Qiagen plasmid mini kit (Qiagen, Stockach, Germany) following the manufacturer's instructions. The pure pL32 and pL41 were quantified using a Nanodrop 2000 microsample spectrophotometer (Thermo Scientific, Wilmington, DE) and used as a positive control to determine the detection limit in the LAMP assay. The plasmid pL32 was also used as the template for the selection of the best primer set. Genomic DNA used in LAMP and qPCR was extracted from* L. interrogans* Copenhageni strain Fiocruz L1-130 by QIAmp DNA Mini Kit (Qiagen, Stockach, Germany) following the manufacturer's instructions. Genomic DNA template from 25* Leptospira*-suspected cultures was also extracted in the same way and used as template in both LAMP and PCR as described below. The genomic DNA from other bacteria (*O. tsutsugamushi, R. typhi, R. conorii, R. rickettsii, C. burnetii,* and* B. bacilliformis*), whose symptoms are commonly mistaken for leptospirosis, were used for cross-reactivity study.

### 2.3. Mimicked Blood Samples

Normal human plasma (SeraCare, Milford, MA) spiked with* L. interrogans* genomic DNA were prepared. A total of 200 *μ*L spiked plasma samples were used for DNA extraction using QIAmp DNA Mini Kit and the extracted DNA was eluted with 20 *μ*L elution buffer. Three independent extractions were performed. Each DNA sample was tested in duplicate in three independent qPCR runs.

### 2.4. LAMP Reaction

LAMP reactions were carried out as described previously [11]. Briefly, a 25 *μ*L reaction mixture contained 1.6 mM of each FIP primer and BIP primer, 0.8 mM of each LF primer and LB primer, 0.2 mM of each F3 primer and B3 primer, 20 mM Tris-HCl (pH 8.8), 10 mM KCl, 8 mM MgSO_4_, 10 mM (NH_4_)_2_SO_4_, 0.1% Triton X-100, 0.8 M betaine (Sigma-Aldrich, St. Louis, MO), 1.4 mM dNTP mixture (New England Biolabs, Beverly, MA), 8 U* Bst* DNA polymerase (New England Biolabs, Beverly, MA), and 5 *μ*L of DNA template. The optimal reaction temperature of 63°C was experimentally determined by varying the temperature from 58 to 63°C. The reaction mixture was incubated for 60 min. Each reaction was terminated by adding 5 *μ*L of 10X BlueJuice (Invitrogen, Carlsbad, CA) for gel detection. The reaction products were examined by electrophoresis on a 2% agarose gel stained with a 1 : 10,000 dilution of GelRed (Phenix Research Products, Asheville, NC). Other detection methods involved inclusion of dyes before amplification, such as addition of HNB [18] into the reaction to enable direct visual detection or inclusion of SYBR green to detect the reaction products by a UV light.

### 2.5. Quantitative Real Time PCR

Quantitative real time PCR was performed to compare and to confirm the sensitivity of the LAMP assay. Serial dilutions of pL32 plasmid were used to obtain the standard curves to determine the copy number of the purified* L. interrogans* Copenhageni strain Fiocruz L1-130 genomic DNA or to determine the limit of detection after the extraction of DNA from spiked samples. ABI 7500 Fast Real Time PCR system (Applied Biosystems, Foster City, CA) was used to perform the qPCR and analyze the amplification data. F3 and B3 primers of primer set L32-5 were used to determine the copy number of the genomic DNA of* L. interrogans* present. The total volume of each reaction was 20 *μ*L. Each reaction mixture contained 0.5 *μ*M of forward primer F3, 0.5 *μ*M of reverse primer B3, 1X RT2 SYBR green qPCR Mastermix (SA-Biosciences, Frederick, MD), and 5 *μ*L of DNA template. An initial 10-minute activation step at 95°C was followed by 40 cycles of 95°C for 15 seconds, 60°C for 1 min, and a melting curve determination cycle.

### 2.6. Leptospiral Species

25* Leptospira*-suspected human specimens were collected and the organisms were cultured from blood using EMJH media in Siriraj Clinical Research Center at Mahidol University in Bangkok, Thailand. The DNA from these cultured samples were extracted using QIAmp DNA Mini Kit. To identify the species of* Leptospira* in each specimen, species-specific primers targeting the* ompL1* gene sequence were used in a PCR assay previously described by Reitstetter [19].

## 3. Results

### 3.1. Selection of Best Primer Sets

Among the six primer sets designed by the LAMP primer designing software, primer sets L32-3 and L32-5 performed the best at 63°C. Both primer sets were able to detect 25 copies of pL32 and primer set L32-1 detected 50 copies of pL32 (see Figure S1 in Supplementary Material available online at http://dx.doi.org/10.1155/2015/147173). Primer sets L32-2, L32-4, and L32-6 did not work at all (data not shown). Primer set L41 was able to detect 50 copies of pL41 (Figure S2) very similar to the previous report at approximately 100 copies [17]. Both primer sets L32-3 and L32-5 were able to detect 25 copies of genomic DNA (Figure S3). The sensitivity of the assay was improved to 12 copies of genomic DNA by combining primer set L32-5 targeting* lipL32* and the primer set targeting* lipL41*(Figure S4). Therefore, the combination of primer sets L32-5 and L41 was used for the rest of the experiments. DNA sequence analysis of the 270 bp region of* L. interrogans* serovar Copenhageni strain Fiocruz L1-130 where the L32-5 LAMP primer set was located showed greater than 99% sequence identity to pathogenic species* L. interrogans*,* L. kirschneri*,* L. borgpetersenii*, and* L. noguchii*. The same region has 96% and 92% sequence identities to pathogenic species* L. weilii* and* L. santarosai*, respectively.

### 3.2. Specificity of LAMP

The specificity of the LAMP assay was evaluated by using the genomic DNA from other bacteria whose symptoms are commonly mistaken for leptospirosis. LAMP reactions containing different strains of* O. tsutsugamushi* (Karp, Kato, Gilliam, and TA763),* R. typhi*,* R. Conorii*,* R. rickettsia*,* C. burnetii,* and* B. bacilliformis* were tested. About 10^6^ copies of each genomic DNA was used as template and all tested negative with the combination of primer sets L32-5 and L41.

### 3.3. Feasibility of LAMP Using Mimicked Blood Samples

To mimic a clinical situation, we tested LAMP using normal human plasma spiked with* L. interrogans* genomic DNA. The LAMP assays had a detection limit of 33 copies per reaction based on standard curves obtained from diluted plasmids (Table 2).

### 3.4. Detection of Different Species of* Leptospira* by LAMP

Out of the 25 samples analyzed, only 18 were PCR positive targeting* ompL1* using species-specific primer sets. The* Leptospira* species identified included nine* L. interrogans* Intergroup A, five* L. interrogans* Intergroup B, two* L. borgpetersenii*, and two* L. weilii.* Further sequencing of the 16s rRNA gene confirmed that these 7 PCR negative samples were not* Leptospira *spp. Thus a total of 18 PCR confirmed* Leptospira*-positive samples and 7 PCR/sequence confirmed* Leptospira*-negative samples were used to evaluate the performance of the LAMP assay blindly. All 18 PCR positive samples were confirmed to be positive by the LAMP assay.

### 3.5. Different Methods for Detection of LAMP Products

**Figure 1:**
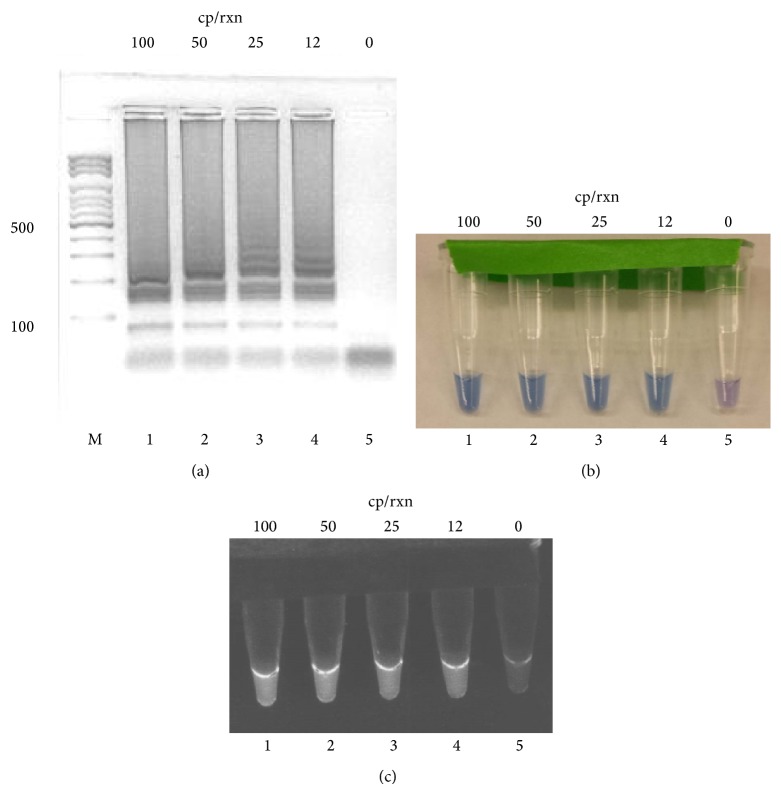
Different methods to detect LAMP products. Reaction products were visualized on GelRed stained gel (a) or visualized directly by including hydroxy naphthol blue (b) or SYBR green (c) in the reaction. A mixture of primer sets L32-5 and L41 was used in the reaction with different copies of genomic DNA. Reactions were carried out at 63°C for 60 min. Marker, 100 bp ladder, lanes 1 to 5, reaction mixture containing 100, 50, 25, and 12, and no copies of genomic DNA.

LAMP reaction products were run on 2% agarose gels stained with GelRed. Positive reactions produced a specific ladder-like pattern (Figure 1(a)). Addition of HNB to the reaction produced a visual color change from purple to blue (as the Mg^2+^ ions in solution were chelated by pyrophosphate ions [13]). The reaction results can be visualized by the naked eye as seen in Figure 1(b). The inclusion of SYBR green in the reaction mixture to detect the reaction products by UV light was also tested (Figure 1(c)). Both alternative methods allow the examination of the results without opening the tube and require no additional process.

## 4. Discussion

Leptospirosis is the most widespread zoonotic disease in the world. Human infection results from contact with water containing* Leptospira* spp. Because the clinical presentation of leptospirosis is very similar to malaria, scrub typhus, and dengue, it is not possible to reliably predict the cause of infection based on the clinical signs and symptoms [20]. Several PCR and qPCRs have been developed for the detection of* Leptospira *spp. [21, 22]. Although PCR and qPCR have advantages with respect to quantification, control of contamination, and sensitivity, both methods require specialized equipment. LAMP on the other hand is a low technology diagnostic tool for resource-limited setting. The test results can be determined by simple visual discrimination.

Previously, Lin et al. [17] reported a LAMP method targeting the* lipL41* gene of* L. interrogans*. In their study, the LAMP products were examined by electrophoresis on a stained agarose gel and the detection limit was approximately 100 copies per reaction. Later on, several LAMP assays targeting the 16S ribosomal RNA gene were also developed [23–25]. Sonthayanon et al. [23] were able to detect both pathogenic and intermediate group* Leptospira* species. The results of LAMP reaction were determined on the basis of detecting a white precipitate with the naked eye after centrifugation. Their results were confirmed by electrophoresis on a stained agarose gel. They demonstrated a lower limit of detection of 10 genome equivalents (20 copies) per reaction versus 100 copies per reaction by earlier method. However, their amplification step takes as long as two hours. With primers specific for pathogenic* Leptospira*, Koizumi et al. [24] were able to detect 10 genome equivalents per reaction by UV fluorescence. They also described that using heat-denatured DNA improved the assay sensitivity to 2 genome equivalents per reaction. The same effect was observed when we developed a LAMP assay for detection of* O. tsutsugamushi* [26]. When DNA was denatured, the copies of exposed single stranded DNA increased which allowed more primers to anneal to the DNA to start a LAMP reaction. We anticipate that by heating the template we will further improve our assay's sensitivity similarly. Suwancharoen et al. [25] were able to detect 10 and 100 copies per reaction, respectively, for serovars Tarassovi and Icterohaemorrhagiae by eye with 90 min incubation time. The LAMP assay that we have developed is as sensitive as the best method mentioned above without heat-denaturing the DNA. The incubation time is shorter than several of the previous methods and most importantly there is no need for centrifugation or UV fluorescence for determination of results. All we need is a heat source to provide a constant temperature at 63°C for 60 min and the results can be read by eye without other instruments.

## 5. Conclusions

Diagnostic testing, in particular early detection, is critical for leptospirosis, as most infected individuals have nonspecific symptoms that are easily confused with dengue and malaria, which require different treatments. LAMP is an attractive alternative to PCR-based methods for early detection since a thermocycler is not required. A heating block or water bath to maintain a constant temperature around 60°C is all that is required for the LAMP assay, making it particularly suited to resource-limited settings. Our study also presents a variety of methods that not only can detect the LAMP products without opening the reaction tubes to avoid a very common contamination problem but also have a shorter assay time. The advantage of using these detection methods, by naked eye or by a small fluorescence reader, will greatly enhance our ability to quickly diagnose an individual for a* Leptospira* infection. This will allow appropriate treatment of acute leptospirosis in a timely manner. This assay has the potential to be used as a rapid, robust, and easy-to-perform assay in the endemic regions. In the future, we would like to prepare the lyophilized LAMP reagents to avoid the requirement of cold chain. Using a stable lyophilized reaction mixture will be a perfect assay to be used in resource-limited settings where leptospirosis is endemic.

## Supplementary Material

Evaluation the performance of six software designed L32 primer sets and finding the best primer sets combination.

## Figures and Tables

**Table 1:** List of primer sequences for LAMP primer sets targeting *lipL32*.

L32-5-F3	TCTATGTTTGGATTCCTGCC
L32-5-B3	ATCGTCACCATCATCATCATC
L32-5-FIP	CGCTTACTAAGTCTCCGTCGCGTAATCGCTGAAATGGGAGT
L32-5-BIP	GCGGCTACCCCAGAAGAAAAGCATAATCGCCGACATTCT
L32-5-LF	CTCACCGATTTCGCCTGT
L32-5-LB	TGCCACATTGGTTTGATACTTG

**Table 2:** Detection limit in normal human plasma spiked with genomic DNA.

Starting material (cp/mL)	2000 (1111)^a^	1000 (555)	500 (278)	200 (111)	0
200 *μ*L for extraction (cp)	400	200	100	40	0
Elute in 20 *μ*L (cp/*μ*L)^b^	20	10	5	2	0
Add 5 *μ*L in LAMP (cp)	100	50	25	10	0
qPCR quantification^c^ (cp)	72 (65~84)	33 (28~40)	12 (8~20)	ND	ND^d^
LAMP result	pos.	pos.	neg.	neg.	neg.

^a^Estimated cp per mL of whole blood, as plasma constitutes 55% of the total blood volume.

^b^Assuming 100% recovery for the extraction steps.

^c^Copy numbers are based on standard curves obtained from diluted pL32 plasmid. They are the average of three independent experiments (range of detection).

^d^ND: not detected.
