# Supplementary material for: Highly Sensitive Loop-Mediated Isothermal Amplification for the Detection of Leptospira
Source: Int J Bacteriol. 2015 Jan 27;2015:147173. doi: 10.1155/2015/147173 (PMC4745460; doi:10.1155/2015/147173)
Supplement: Supplementary file 1 — Evaluation the performance of six software designed L32 primer sets and finding the best primer sets combination. [file 147173.f1.pdf]

Supplementary Figures

S1. LAMP results for the serial dilutions of pL32 plasmid with primer set L32-1 (A), L32-3 (B), L32-5 (C). Reaction mixtures were incubated at 63°C for 60 min. Marker, 100 bp ladder; Lane 1 to 5, reaction mixture containing 100, 50, 25, 12 and no copies of plasmid DNA.

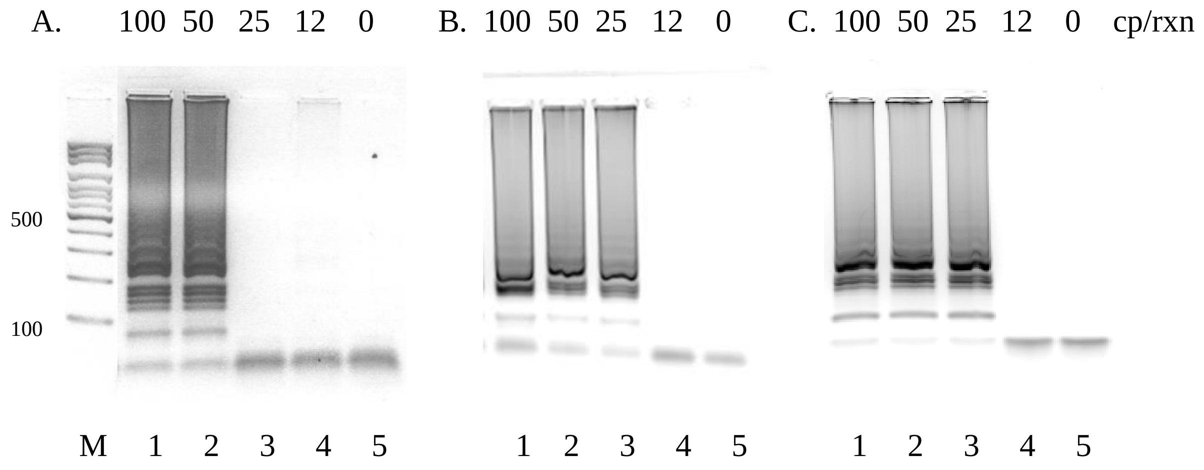

S2. LAMP results for the serial dilutions of pL41 plasmid with primer set L41. Reaction mixtures were incubated at 63°C for 60 min. Marker, 100 bp ladder; Lane 1 to 6, reaction mixture containing 200, 100, 50, 25, 12 and no copies of plasmid DNA.

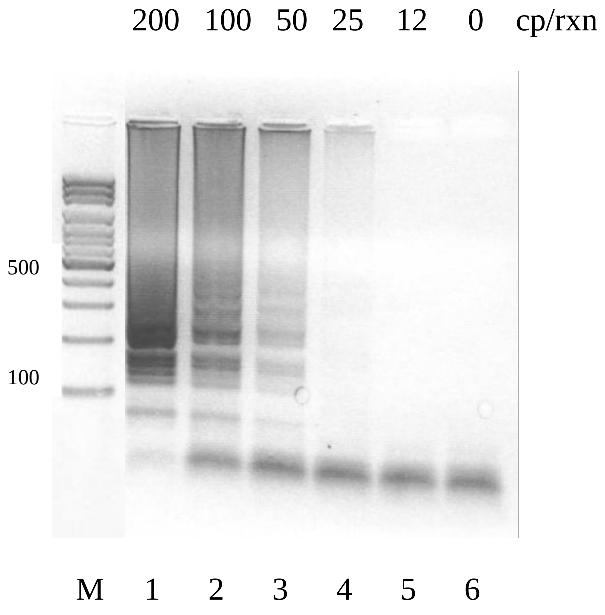

S3. LAMP results for the serial dilutions of *L. interrogans* genomic DNA with primer set L32-3 (A), L32-5 (B). Reaction mixtures were incubated at 63°C for 60 min. Marker, 100 bp ladder; Lane 1 to 5, reaction mixture containing 100, 50, 25, 12 and no copies of genomic DNA. The copy number of the original genomic DNA was determined by qPCR.

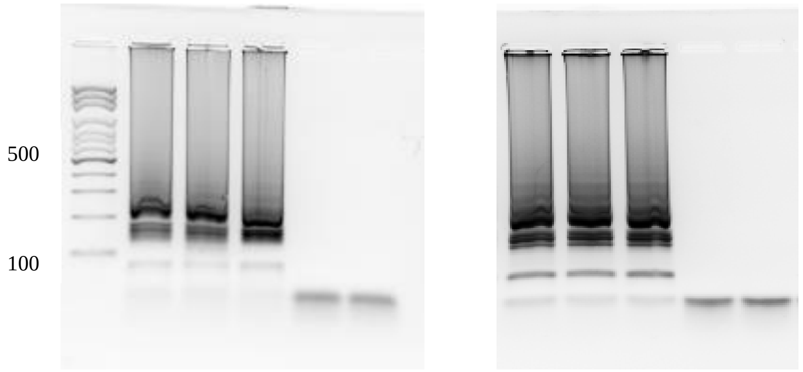

S4. LAMP results for the serial dilutions of *L. interrogans* genomic DNA with combinations of primer set L41 and L32-3 (A), L41 and L32-5 (B). Reaction mixtures were incubated at 63°C for 60 min. Marker, 100 bp ladder; Lane 1 to 9, reaction mixture containing 100, 100, 50, 50, 25, 25, 12, 12 and no copies of genomic DNA.

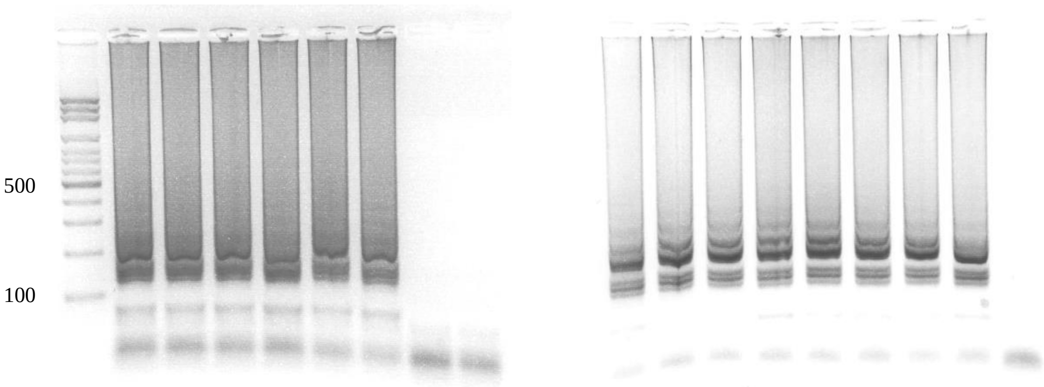

Table S1. List of other primer sequences for LAMP primer sets targeting *lipL32*

L32-1-F3	TTTGGCTATCTCCGTTGC
L32-1-B3	CGATTACGGCAGGAATCC
L32-1-FIP	GATTGTGTCCTCGCTCAGAACAAAGCATTACCGCTTGTGG
L32-1-BIP	CCAGGGACAAACGAAACCGTAGGCTTTACGTATCCGTAATAGT
L32-1-LF	TTAGGCTTGGCAGACCAC
L32-1-LB	CGTTACTTCCCTACGGATCTG
L32-2-F3	GGATTCCTGCCGTAATCG
L32-2-B3	ATCGTCACCATCATCATCATC
L32-2-FIP	CGCTTACTAAGTCTCCGTCGCATGGGAGTTCGTATGATTTCC
L32-2-BIP	GCGGCTACCCCAGAAGAAAAGCATAATCGCCGACATTCT
L32-2-LF	CTCACCGATTTCGCCTGT
L32-2-LB	TGCCACATTGGTTTGATACTTG
L32-3-F3	TTGGATTCCTGCCGTAATC
L32-3-B3	ATCGTCACCATCATCATCATC
L32-3-FIP	CGCTTACTAAGTCTCCGTCGCGCTGAAATGGGAGTTCGTAT
L32-3-BIP	GCGGCTACCCCAGAAGAAAAGCATAATCGCCGACATTCT
L32-3-LF	CTCACCGATTTCGCCTGT
L32-3-LB	TGCCACATTGGTTTGATACTTG
L32-4-F3	TCTCTATGTTTGGATTCCTGC
L32-4-B3	ATCGTCACCATCATCATCATC
L32-4-FIP	CGCTTACTAAGTCTCCGTCGCCGTAATCGCTGAAATGGGA
L32-4-BIP	GCGGCTACCCCAGAAGAAAAGCATAATCGCCGACATTCT
L32-4-LF	CTCACCGATTTCGCCTGT
L32-4-LB	TGCCACATTGGTTTGATACTTG

L32-6-F3	CTGCCGTAATCGCTGAAA
L32-6-B3	ATCGTCACCATCATCATCATC
L32-6-FIP	CGCTTACTAAGTCTCCGTCGCTGGGAGTTCGTATGATTTCC
L32-6-BIP	GCGGCTACCCCAGAAGAAAAGCATAATCGCCGACATTCT
L32-6-LF	CTCACCGATTTCGCCTGT
L32-6-LB	TGCCACATTGGTTTGATACTTG
